# Which is the best way to treat a stone on a flexible ureterorrenoscopy? | *Opinion: Fragmentation*

**DOI:** 10.1590/S1677-5538.IBJU.2017.05.02

**Published:** 2017

**Authors:** Alex Meller

**Affiliations:** 1 Divisão de Endourologia e Litíase Renal e Disciplina de Urologia – Universidade Federal de São Paulo, UNIFESP/EPM, SP, Brasil

**Keywords:** Calculi, Lasers, Solid-State, Kidney

Since the introduction of Holmiun YAG (Ho-YAG) laser to treat kidney or ureteral stones, a dramatic change in techniques of stone treatment has occurred, especially how to adjust the ideal laser setting to achieve ideal fragmentation.

First reports of Ho-YAG laser clinical application had been focused on tissue cutting or destruction (1), but few years later, the ability to fragment stones through a thermal mechanism was demonstrated (2). The laser emission superheats water surrounding the laser fiber tip, thus creating a microscopic vaporization bubble that is able to destabilize or vaporize tissue or a stone. Based on this mechanism, the ideal energy setting to treat a stone had been discussed and evaluated.

Nowadays, the concept of dusting and fragmentation settings are very well documented, but the studies failed to prove which technique is superior. In dusting technique, the goal is to pulverize a stone creating small particles and dust (< l–2mm in size) to theoretically enable spontaneous passage of the small particles. In fragmentation and retrieving technique, stones can be fragmented into pieces (l-4mm) depending on the access sheath lumen used and will be extracted with a grasper or basket thoroughly cleaning the collecting system or ureter.

There are three different parameters related to laser settings: energy, frequency and pulse. The laser power delivered in Watts is obtained multiplying energy X frequency, enabling a lot of combinations. Older publications demonstrated that increasing pulse energy (Joules) will increase fragmentation rate (3) but frequency was not evaluated. An elegant study *in vitro* from Kronenberg et al. (4) evaluated different settings and its stone ablation rate, using pulse energy and frequency. It has concluded that stone ablation increased while increasing pulse energy, regardless of frequency or total power (Watts), but this combination can produce bigger fragments (5) and its removal could be time consuming. Conversely, increases in frequency will slower fragmentation rate (6) at the same pulse energy, but it will produce smaller fragments (5) that can be cleared spontaneously, avoiding the need to retrieve all particles.

In newer lithotripter models, the urologist can adjust different pulse durations, i.e., the traditional short-pulse mode (330μs) or a long-pulse mode (650-1250μs) (7). Conceptually, pulse duration is the period of time that energy will be delivered during a single laser pulse, which can modify stone ablation rate. Preliminary studies showed that short-pulse mode is more ablative than the long-pulse mode (7), but at cost of more retropulsion effect (8). Others suggested that short-pulse mode could produce bigger fragments than long-pulse mode, but it couldn't be proved experimentally (9). More studies are needed to define the ideal pulse duration to achieve best performance, but in the author's opinion, long-pulse mode could perform better in softer stones and, theoretically, produce a better dust effect with less retropulsion.

The questions that emerge from these two concepts (dusting or basketing) are related to clinical outcomes, auxiliary procedures, absence of stone specimen to be analyzed and costs.

Comparative studies about clinical outcomes are rare. The only published study that compared dusting versus basketing for ureteral stones randomized 60 patients (30 in each arm) to undergo active retrieval or wait for spontaneous passage after laser lithotripsy with a semirigid ureteroscope. The primary study outcome was difference in unplanned medical or emergency room visits up to 30 days after surgery. Secondary outcomes evaluated included need for analgesia, need for auxiliary procedures, stone free rate and difference in hospitalization. Overall, the dusting group had a higher rate of unplanned visits than the basketing group (30% versus 3%, OR 12.4, 95% CI 1.8-80.3, P=0.01) (10). Other parameters as stone free rate, re-hospitalization rates, need for analgesia and need for auxiliary procedures hadn't shown significant statistical differences, but all of them tend to be worst in dusting group. Despite its randomized design, this study has many problems (multiple surgeons, variable laser settings, exclusion of patients using ureteral stents, etc.), compromising the outcomes.

Chew et al. are prospectively evaluating both strategies for renal stones (5-20mm) in a multicenter study (EDGE consortium) involving 8 high volume tertiary stone centers. They enrolled 152 patients (basketing = 82, dusting = 70) and followed then for 3 months. The preliminary results evaluated stone free rates as primary outcome, and presence of residual fragments, readmission rate, duration of procedure, amount of energy used and need for additional procedures. Stone free rates at initial follow up were 86.3% in the basketing group versus 59.2% in the dusting group, with fewer patients who underwent basketing having symptoms from residual fragments or requiring secondary interventions. Besides the higher presence of residual fragments in the dusting group, the readmission rate was not statistically different in both groups in a short-term follow-up. The dusting approach results in shorter operative time and lower use of ureteral access sheath (UAS) than basketing group. However, there are problems in this important study: the short follow up duration, multiple surgeons and use of KUB and ultrasound to evaluate residual fragments could bias its results.

The natural history of residual fragments was evaluated in several studies. A retrospective study from Chew and associates evaluated 232 subjects with residual fragments 12 months after ureteroscopy between 2006 and 2013. The stone event rate was 44%, where 29% required intervention and 15% experienced complications without intervention. Fragments larger than 4mm were more likely to grow with time (p<0.001) and were associated with more complications (p=0.039). Fragments larger than 2mm are more likely to grow (p<0.001) but were not associated with complications or re-intervention. Re-intervention rates were predictable based on fragments size (p=0.017) (11). Other studies demonstrated that presence of hydronephrosis and lower-pole stones are predictors of failure on residual fragments clearance (12, 13).

These studies highlighted the importance to achieve the real stone free status to prevent re-intervention or stone re-growth, but failed to prove it statistically. But, between 20 to 30% of patients with residual fragments will need an additional procedure and this fact cannot be underestimated.

Basketing technique needs to be performed with UAS elevating the risks of ureteral injury (14), but it also means that intrarenal pressure will be lower during flexible procedure (15), preventing risks of SIRS. This is a controversial point, where cost and benefits still has to be evaluated.

Additionally, immediate costs will be higher in basketing technique procedure where use of a basket device and UAS is mandatory. But none of studies evaluated the global cost including the higher rate of unplanned emergency room visits, additional procedures and re-growth of residual fragments in the patients submitted to dusting technique. Then, more sophisticated cost evaluation studies should be done to answer this question.

Other criticism of dusting technique is the lack of specimen to be analyzed. Assuming that stone disease has a high recurrence rate, with at least 50% of individual experiencing another stone in 5 years, medical prevention is the key to minimize this condition. Knowing the stone composition is one of the major points to prevent recurrence.

New high power lasers (120W) developed for prostate ablation have been used for stone treatment a few years ago. Since then, dusting technique seems to be more efficient and widespread the idea that truly pulverizes the stone. Few studies tested this new technology and none compared head to head basketing and high frequency laser technique. Emiliani and cols, tested different frequencies and pulse energy in an experimental study. They demonstrate that high power was more efficient in reduction of stone volume than lower frequencies (OR 1.14, 95% CI 1.09 − 1.20) (16) This new concept could improve dusting results and establish a new paradigm, but the laser unit is very expensive, making it difficult to be used worldwide. Maybe in the future the costs will lower and availability of high power lasers will become the new standard.

Besides all literature, a subjective parameter has to be cited. The perfect dusting technique to produce smaller fragments as possible (real dust) may be less related to laser settings and more dependent on the surgical technique. Studies comparing dusting (high frequency/low energy) versus fragmentation (low frequency/high energy) settings used irregular hand-held approaches or automated testing systems, which failed to reproduce real life situation. The way that urologists approach the stone with the laser, i.e. repeatedly perforating, chipping or fragmenting in comparison with working on its surface, “painting” the stone surface could be the most important factor, and this remains to be investigated.

Finally, the debate still ongoing, but some considerations could be done at that point. Every stone needs a personalized approach, if they are softer, harder, smaller, bigger or located in renal collecting system or ureter. It seems reasonable that a harder stone will need more energy and fragmenting should perform better, but if it is too big, it could produce a prohibitive amount of fragments. On contrary, in softer stones, a technically correct dusting could pulverize the stone and achieve good stone free rates, but sometimes, smaller stones are technically challenging to be pulverized. The urologist must be aware of the fragments size produced during lithotripsy and adjust settings accordingly it in a dynamic way.

Our patients wish to be free of stones, with less pain, less additional procedures and lower complications. Then, in our opinion, the best approach is to reduce stone burden in bigger stones (over 10mm) with dusting technique and fragmenting the residual stone (less than 10mm) in pieces to be retrieved, combining both techniques. When treating smaller stones (less than 10mm), fragmenting and basketing seems to be preferable to meet the patient goal, the higher immediate stone free rate.
